# The Unresolved Role of Interferon-λ in *Asthma Bronchiale*

**DOI:** 10.3389/fimmu.2017.00989

**Published:** 2017-08-15

**Authors:** Nina Sopel, Andreas Pflaum, Julia Kölle, Susetta Finotto

**Affiliations:** ^1^Department of Molecular Pneumology, Friedrich-Alexander-Universität Erlangen-Nürnberg, Universitätsklinikum Erlangen, Erlangen, Germany

**Keywords:** asthma, rhinovirus, interferon, exacerbation, epithelial cell

## Abstract

*Asthma bronchiale* is a disease of the airways with increasing incidence, that often begins during infancy. So far, therapeutic options are mainly symptomatic and thus there is an increasing need for better treatment and/or prevention strategies. Human rhinoviruses (HRVs) are a major cause of asthma exacerbations and might cause acute wheezing associated with local production of pro-inflammatory mediators resulting in neutrophilic inflammatory response. Viral infections induce a characteristic activation of immune response, e.g., TLR3, 4, 7, 8, 9 in the endosome and their downstream targets, especially MyD88. Moreover, other cytoplasmic pattern recognition molecules (PRMs) like RIG1 and MDA5 play important roles in the activation of interferons (IFNs) of all types. Depending on the stimulation of the different PRMs, the levels of the IFNs induced might differ. Recent studies focused on Type I IFNs in samples from control and asthma patients. However, the administration of type I IFN-α was accompanied by side-effects, thus this possible therapy was abandoned. Type III IFN-λ acts more specifically, as fewer cells express the IFN-λ receptor chain 1. In addition, it has been shown that asthmatic mice treated with recombinant or adenoviral expressed IFN-λ2 (IL–28A) showed an amelioration of symptoms, indicating that treatment with IFN-λ might be beneficial for asthmatic patients.

## Asthma Bronchiale

Currently, there are about 300 million people worldwide suffering from the chronic airway inflammatory disease *Asthma bronchiale*. There are two forms of asthma: intrinsic, or non-allergic and allergic, or atopic asthma, although both forms can co-exist in some patients. Intrinsic asthma often appears later in life and the causes are viral infections of the lower airways or irritants such as cold air, cigarette smoke, or stress whereas, allergic asthma affects prevalently children and about one half of adult asthmatic subjects. Here, the triggers for the disease are usually innocuous substances, e.g., proteins from plant or tree pollen, house dust mite, or animal dander (1–3).

The symptoms of asthma comprise airway hyper-responsiveness (AHR), mucus hyperproduction, reversible airway obstruction, airway remodeling, and in case of allergic asthma, high serum IgE levels. Together, these symptoms cause recurrent shortness of breath, wheezing, and chest tightness due to a narrowing of the airways (2, 3).

## Immunology of Asthma

The asthmatic airways host dysregulated immune reactions as a pathological response to an otherwise innocuous allergen. The dysregulated immune responses seen in asthma are mediated by both cells of the innate and adaptive immune system. In a first step, dendritic cells (DCs), located below the airway epithelium, sample allergens from the airway lumen, process these allergens into smaller peptides, which are then presented to naïve T cells in the regional lymph nodes. Here, in the presence of interleukin 4 (IL-4), antigens activate T helper cells and facilitate their differentiation into T helper cells type 2 (Th2), which migrate into the airway mucosa, where they release high amounts of the classic Th2 cytokines like IL-4, IL-9, and IL-13 as well as the cytokines with a common beta chain involved in granulocytes development such as IL-3, IL-5, and granulocyte macrophage colony-stimulating factor (GM-CSF). These mediators have downstream effects on other immune cells, for example IL-3 differentiates mucosal mast cells which are present into the or beneath the bronchial epithelial at the site of inflammation, while GM-CSF in concert with IL-3 and IL-5 favors recruitment, maturation, and survival of eosinophils which are the predominant type of cells around the asthmatic bronchi (4). Eosinophils then release cationic proteins responsible of the cytotoxic effect on the epithelial cells observed in asthma (5). In B cells, the class-switch recombination of immunoglobulins is based to IgE production by IL-4. IgE antibodies bind to the high affinity IgE receptor expressed on, e.g., mast cells. After repeated allergen challenge, IgE cross-links the high affinity IgE receptor bound on the cell surface of mast cells and activate downstream the release of preformed broncho-constrictive, pro-inflammatory mediators such as histamine, leukotrienes, cytokines, or chemokines (2, 3).

Moreover, it has been recently demonstrated that exposure to aeroallergens results in increased differentiation and proliferation of Th17 cells producing IL-17A, resulting in neutrophils accumulation in the airways which is a signature of severe asthma (6, 7). IL 17A is also released by cells of the innate immune system like gamma delta cells, which have been shown to be important on the development of asthma (8). Furthermore IL 17A is also involved in mucosal and epithelial host defense against infections and thereby it constitutes an important cytokine mediating antiviral immune responses (9, 10).

Finally, it has been recently demonstrated that allergen can induce AHR without previous systemic sensitization *via* the upregulation of group 2 innate lymphoid cells (ILC-2) that lack antigen-specific receptor and function *via* cytokine signaling (11, 12). In the immunological response to the allergen, ILC2 are positioned downstream of infection or allergen damaged epithelial cells and after activation they produce Th2 cytokines like IL-5, IL-9, and IL-13 *via* ST2 activation and without the need of T cells. ILCs in general have also been involved in clearing infection (13–16). The mechanism involving ILC2 activation occurs *via* IL-33, a cytokine of the IL-1beta family, released by necrotic epithelial or endothelial cells after allergen challenge or virus infection (17, 18). IL-33 is the ligand of ST2, also known as IL-1RL1, and is present on the surface of both Th2, ILC2, mast cells (11). For this reason, these newly discovered cells play an important role in the asthmatic airways to resolve the inflammation and in clearing infections.

Viral infections, especially with rhinovirus (RV), play a key role in the development of asthma and asthma exacerbations, particularly in children (19–23). Therefore, the focus of this review article is the role of RV infections in asthma, the downstream interferon (IFN) immune response, with emphasis on type III IFNs, and their potential role as therapeutic agents in asthma.

## RV Structure, Genomic Organization, Replication, and IFN Induction

Human rhinoviruses (HRVs), described first in the 1950s, are the primary causative agent of the common cold (24). HRV infections are concomitant with exacerbations of chronic pulmonary disease, severe pneumonia in elderly and immunocompromised adults, asthma development as well as serious bronchiolitis in infants and children (25, 26).

Human rhinovirus is a member of the family *Picornaviridae* and the genus *Enterovirus*. More than 150 identified HRV serotypes were divided into the three groups HRV-A, HRV-B, and HRV-C, according to their phylogenetic similarity (27–29).

Human rhinovirus is a non-enveloped positive-sense, single-stranded RNA (ssRNA) virus with a genome size of approximately 7,200 bp and a single open reading frame. The translated polyprotein can be divided into the three regions P1, P2, and P3, whereby the P1 region encodes for the viral capsid proteins VP1, VP2, VP3, and VP4 (termed the protomer). The P2 and P3 regions encode proteins involved in protein processing (2A^pro^, 3C^pro^, 3CD^pro^), genome replication and assembly (2B, 2C, 3AB, 3B^VPg^, 3CD^pro^, 3D^pol^) (26, 30–32).

The virions consist of 60 copies each of the four capsid proteins building an icosahedral symmetric capsid of about 30 nm in diameter. Thereby VP1, VP2, and VP3 create the protein shell, by contrast VP4 is in the inner site of the virus, anchoring the RNA core to the capsid (33–35). Resolution of the structure revealed a prominent, star-shaped plateau, surrounded by a deep depression, also called canyon, which is the site of attachment to the different cell surfaces receptors (36, 37).

The replication of HRV takes place in the cytoplasm of epithelial cells of the lower and upper airways (38). In early HRV life cycle, it attaches to a cell membrane receptor. More than 90% of HRVs (“major” group), interact with the amino-terminal domain of the intercellular adhesion molecule 1 (ICAM-1; CD54) (39–41). The remaining “minor” group binds and enters the cell *via* a member of the low-density lipoprotein receptor family (42, 43), whereas some of the HRVs also use heparan sulfate as an additional receptor (44, 45). Moreover, HRV-C binds to Cadherin-related family member 3 (CDHR3) to enter the cells (38). Once HRV has attached to its cellular receptor, the virus capsid undergoes conformational changes, resulting in the release of the viral RNA into the cytoplasm directly (major group) (46), or by endosomal compartments (minor group) (47, 48). Following the translocation of the RNA into the cytoplasm, the viral genome replicates and translates to generate viral proteins, which are essential for the viral genome replication and the production of new virus particles.

During HRV infection, the virus adheres within 15 min to the cell surface receptors into the respiratory tract, thus the infection occurs very quickly. High-risk individuals for infection are children and elderly and infected individuals, which will experience symptoms within 2 days after infection (49). Moreover, HRV-C specie may be able to cause severe infections (50).

While the virus replicates and spreads, the infected cells secrete inflammatory mediators, such as chemokines and cytokines. DsRNA produced during viral infection induces the host innate immune response. It is recognized and ligated by three pattern recognition molecules (PRMs): toll-like receptor (TLR)-3, which is localized at endosomal and plasma membranes, and cytoplasmic proteins retinoid acid-inducible gene 1 (RIG)-I, and melanoma differentiation-associated gene 5 (MDA-5), which are intracellular receptors for viral short-dsRNA and long-dsRNA, respectively (51–53).

## Microbial Induced IFN Immune Responses

**Figure 1:**
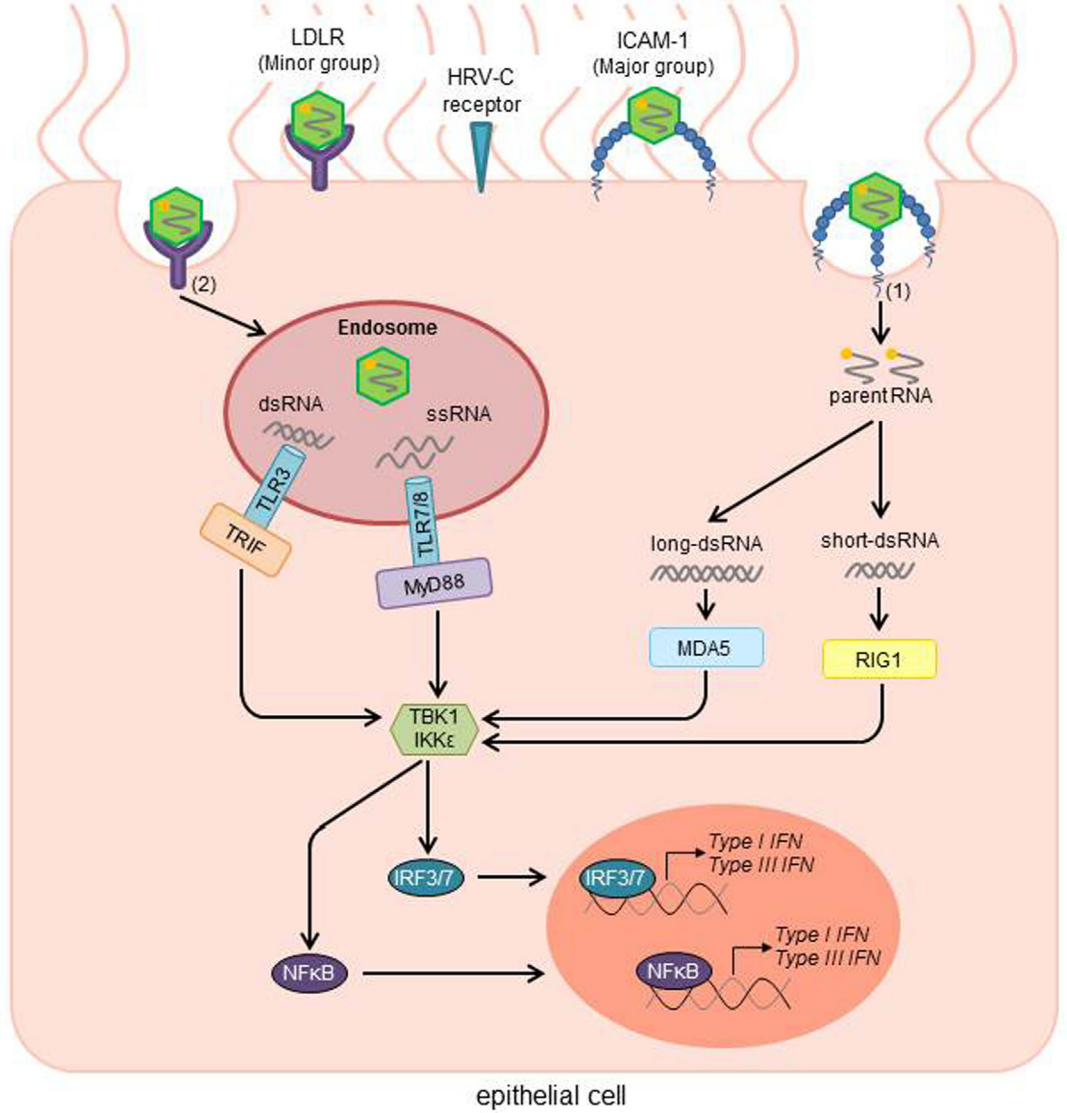
HRV activated signal transduction pathways of the innate immune response after infection of airway epithelial cells. Depending on the receptor type, virus uptake occurs directly without a need for any cellular machinery [Major group; (1)] or *via* clathrin-dependent endocytosis [Minor group; (2)]. After uncoating, viral RNA activates cytosolic and endosomic pattern recognition molecules (PRMs). While the retinoid acid-inducible gene 1 (RIG1) recognizes short viral dsRNA, the melanoma differentiation-associated gene 5 (MDA5) binds to long dsRNA. In the endosome, viral dsRNA and ssRNA are recognized by the Toll-Like receptors TLR3 and TLR7/8, respectively. After recognition of the viral RNA, TLR7/8 then stimulates the adaptor protein myeloid differentiation factor 88 (MyD88), whereas TLR3 activates the Toll/IL-1 receptor domain-containing adaptor inducing IFNβ (TRIF). In further steps, TRIF, MyD88, MDA5, and RIG1 activate the TANK-binding kinase-1 (TBK1) and/or the inducible IκB kinase (IKK). Those factors subsequently induce the interferon regulatory factors 3 and 7 (IRF3/7), as well as NFκB as transcription factors for type I and III IFNs. HRV, human rhinovirus; ICAM-1, intercellular adhesion molecule 1; LDLR, low-density-lipoprotein receptor; RNA, ribonucleic acid; ssRNA, single-stranded RNA; dsRNA, double-stranded RNA; IFN, interferon [adapted from Ref. (26, 55)].

The PRMs MDA-5 and RIG-1 are known to induce type I IFN expression by sensing viral dsRNA in the cytosol (Figure 1). RIG-1 has been shown to be involved in virus-induced type III IFN production. Some studies showed type III IFN expression in DCs and monocytes after stimulation with bacterial lipopolysaccharide (LPS), transmitted by TLR4, indicating that also bacterial infections induce the production of these cytokines (54, 55). Based on the type of receptor they signal through, IFNs, cytokines named after the ability to “interfere” with viral replication, are divided into three major groups, some of which consist of subgroups, called type I, containing IFN-α, IFN-β, IFN-ε, IFN-κ, and IFN-ω, type II, including IFN-γ, and type III, consisting of IFN-λ (56, 57). In 2003, two independent research groups found the three highly related cytokines and while one group attributed them to the IFNs and called them IFN-λ1, IFN-λ2, and IFN-λ3, creating the type III IFNs, the other group described them as IL29, IL-28A, and IL-28B, respectively (58, 59).

Type III IFN is described to not be expressed continuously but to be co-induced with type I IFNs in different cells by various human viruses, as well as by ligands of TLR3, mimicking viral infection (synthetic dsRNA; polyI:C), TLR4 (house dust mite antigen), TLR9 (unmethylated DNA rich in CpG motifs), and TLR7 and TLR8 (guanosine or uridine-rich ssRNA or resiquimod) (55, 58, 60).

The promoters of the *IFNL* genes contain binding sites for the transcription factors NF-κB (nuclear factor κB) and AP-1 (dimerizing with other transcription factors like FOS, JUN, ATF, and MAF), and several virus response elements are the binding sites of IFN regulatory factors (IRFs). The transcription of the type I *IFN* genes is regulated by these factors as well. Furthermore, the NF-κB and IRF pathways were shown to be very relevant to the transcriptional regulation of the *IFNL* genes (60).

While the transcription of human *IFNL1* and *IFNB* genes seem to be controlled similarly by either IRF3 or IRF7, *IFN-L2/3* genes share the dependency on IRF7 with most *IFNA* genes. IRF3 is produced continuously and ubiquitously in cells and increases the expression of the *IFNB* and *IFNL1* genes on recognition of viral entry. In addition to IRF3, *IFNA* and *IFNL2/3* genes need IRF7, which is upregulated in response to IFNs and not continuously expressed in most cell types otherwise. This upregulation in cells can, in humans, be primed by both IFN-β and IFN-λ1 in response to viral induction, leading to IFN-α and IFN-λ2/3 being produced. It has been shown that, as described for type *I IFN* genes, *IFNL* genes utilize a positive feedback mechanism in their expression (60).

After entering the cell, some viruses leave the cytoplasm to enter the endocytic compartments. Depending on the compartment, different PRMs are responsible to detect the antigen, using various ways of inducing IFNs. TLR7, TLR8, and TLR9 activate the adaptor protein myeloid differentiation factor-88 (MyD88), opposed to TLR3 using the Toll/IL-1 receptor domain-containing adaptor inducing IFN-β (TRIF). MyD88, TRIF, RIG-1, and PKR each activate the production of type I IFN by utilizing the TANK-binding kinase-1 (TBK1) and/or the inducible IκB kinase (IKK-i), which activate the transcription factors IRF3 and IRF7, inducing the transcription of type I and type III IFN (Figure 1) (57).

TLR7, TLR8, and TLR9 use MyD88, activating NF-κB and the mitogen-activated protein kinases (MAPKs), as well as c-Jun N-terminal kinase (JNK), p38, and extracellular signal-regulated kinase (ERK). MyD88 then utilizes the serine-threonine kinase IRAK, tumor necrosis factor (TNF) receptor-associated factor-6 (TRAF6) and a MAPK kinase kinase (MAPKKK) called transforming growth factor β-activated kinase (TAK-1), to stimulate the IKK complex. After activation IKK leads to phosphorylation and subsequent degradation of IκB, the release of NF-κB, and induction of NF-κB-dependent genes, such as the pro-inflammatory cytokines IL-1, IL-6, and TNFα. Furthermore, TAK-1 can also activate MKK3 and MKK6, two enzymes upstream of JNK, MAPK, and p38 (57).

## IFN Receptor Signaling and Receptor Distribution

Type I IFNs, which signal through the widely distributed, heterodimeric complex consisting of the two receptor chains IFN-αR1 (IFNAR1) and IFN-αR2 (IFNAR2), whereas type III IFNs use a heterodimeric complex, which consists of the IFN-λR1 chain (IL–28RA) and the shared IL-10R2 chain. The latter receptor chain is also used by the cytokines IL-10, IL-22, and IL-26. The IFN-λR1 chain is encoded on human chromosome 1 and murine chromosome 4, the IL-10R2 chain on chromosome 21 or 16, respectively, in proximity to the IFN-αR 1 and 2 chains [reviewed in Ref. (61)].

**Figure 2:**
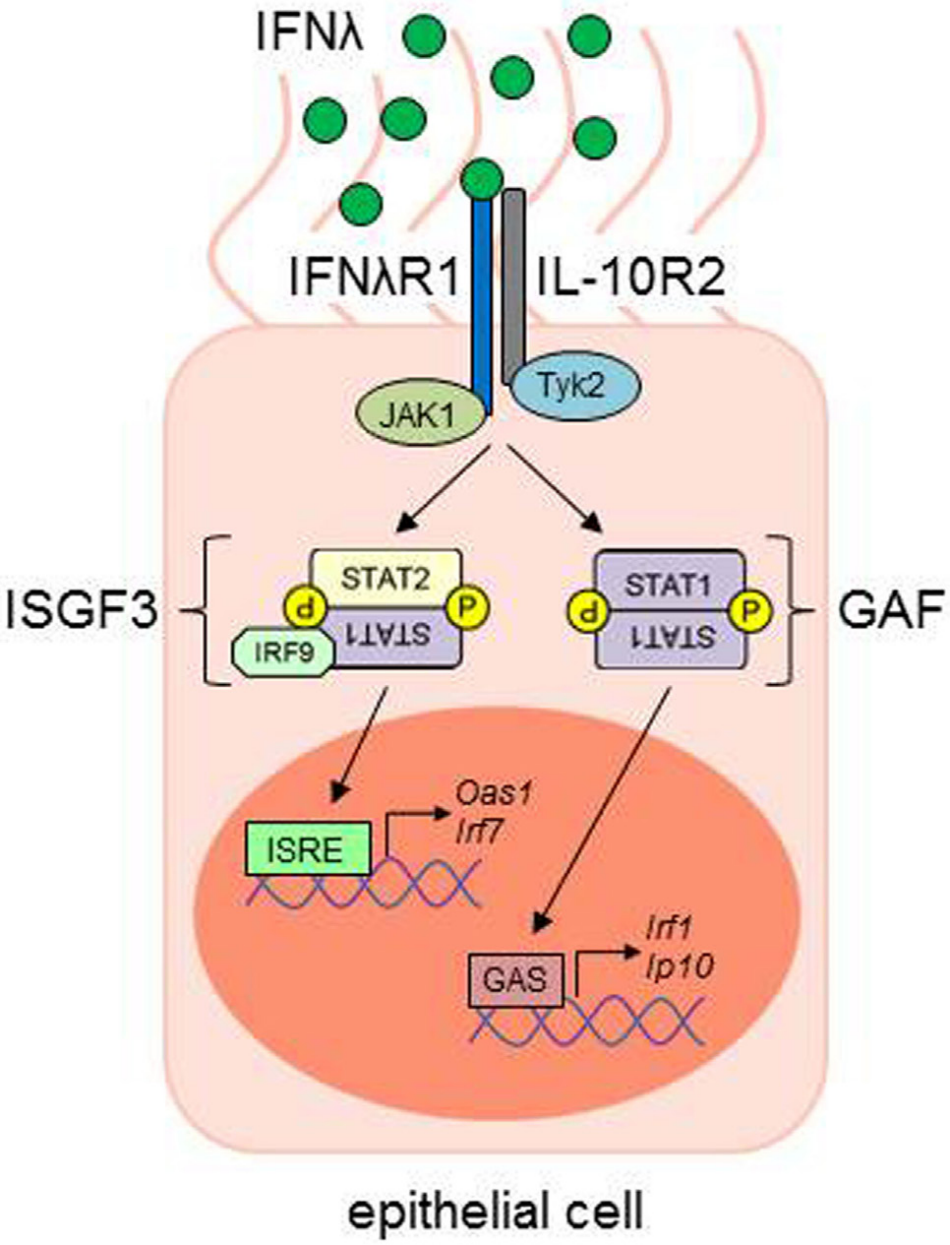
IFN-λ signaling in epithelial cells. IFN-λ binds to its IFNλR1, which leads to a conformational change and the recruitment of the IL-10R2 chain. Receptor-associated kinases (JAK1, Janus kinase 1; Tyk2, tyrosine kinase 2) trans-phosphorylate the respective receptor chains, leading to the phosphorylation and activation of signal transducer and activator of transcription (STAT) proteins. STAT1 and STAT2, together with IFN-regulatory factor 9 (IRF9), build the IFN-stimulated gene factor 3 (ISGF3) transcription factor complex, while the STAT1 homodimer is also named gamma-IFN activated factor (GAF). ISGF3 and GAF complexes are able to translocate to the nucleus and bind to IFN-stimulated response elements (ISRE) or gamma-IFN activation sites (GAS), respectively, in the promoter regions of IFN-stimulated genes (ISG), e.g., *Oas1, Irf1, Irf7*, or *Ip10* [adapted from Ref. (61)]. ISG, interferon-stimulated genes.

When type III IFN binds to its unique receptor chain, IFN-λR1, this facilitates a conformational change causing the recruitment of the second part of the receptor, the IL-10R2 chain. *Via* the receptor-associated Janus kinases JAK1 and Tyk2, docking sites for different STAT proteins are created, e.g., STAT1 and STAT2. Both IFN-αR and IFN-λR signaling lead to the formation of a transcription factor complex, consisting of STAT1, STAT2, and IRF9, the so called IFN-stimulated gene factor 3 (ISGF3). This complex is able to translocate to the nucleus, where it mediates the expression of several IFN-stimulated genes (ISG), by binding to IFN-stimulated response elements (ISRE) in the promoter regions of the ISGs (Figure 2) [reviewed in Ref. (61)].

**Figure 3:**
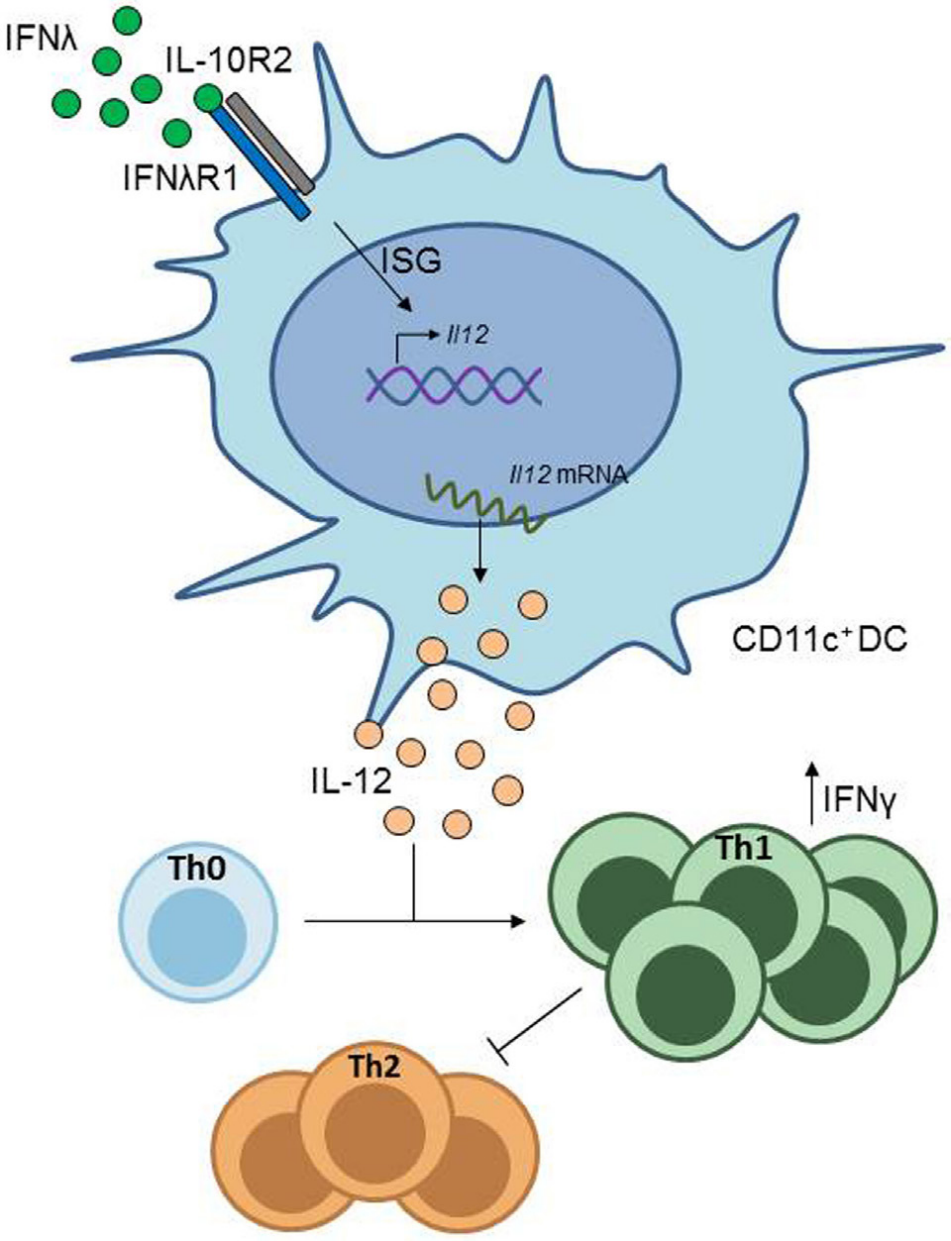
IFN-λ signaling in CD11c^+^ dendritic cells (DCs). CD11c^+^ DCs express the IFN-λ receptor. Stimulation of CD11c^+^ DCs with IFN-λ induces IL-12 production in these cells, which drives Th1 differentiation from naïve CD4^+^ T cells, resulting in inhibition of Th2 cell differentiation.

IFN-αR is expressed on a broad range of cells, e.g., primary fibroblasts, murine splenocytes, or human endothelial cells from the umbilical cord vein (HUVEC), while the action of IFN-λ is more limited. Here, it has been described that on epithelial cells in the respiratory, gastrointestinal and urogenital tract IFN-λR is broadly expressed, whereas the response of PBMCs and cells isolated from bone marrow show only modest responses when stimulated with IFN-λ [reviewed in Ref. (61, 62)]. In addition, it has been shown that CD11c^+^ DCs isolated from the lung (Figure 3), as well as alveolar macrophages and bone marrow-derived DCs express the IFN-λR1, while inflammatory cells in the peribronchial region do not express this receptor subunit. In this study, it was also observed that CD4^+^ T cells isolated from lung or spleen and *in vitro* differentiated Th1, Th2, and Treg cells do not significantly express IFN-λR1 and therefore are not affected by IFN-λ treatment (63). Furthermore, other groups described a lack of IFN-λR expression on leukocytes, primary fibroblasts, HUVECs, and murine splenocytes (61). Interestingly, the shared IL-10R2 chain is also expressed rather broadly, so that the limited action of IFN-λ is restricted by the selective expression of the IFN-λR chain (62).

## Immunological Role of Type III IFN in Asthma

It has been previously proposed that in asthmatic patients, the IFN response to RV is impaired. *In vitro* infection of primary human bronchial epithelial cells (HBEC) isolated from asthmatic or healthy control subjects with the major group RV subtype RV16, revealed increased viral replication and delayed cell death of infected cells in cells from asthmatics. In addition, these cells released decreased amounts of the type I IFN IFN-β, while treatment of cells from asthmatics with exogenous IFN-β enhanced apoptotic cell death and reduced viral replication, especially when cells were pre-treated with IFN-β before RV infection (64). In a following study extending these findings, it was shown that in the human bronchial epithelial cell line BEAS-2B and in peripheral blood mononuclear cells (PBMC) from healthy volunteers, infection with RV16 induced Type III IFN expression both on mRNA and protein level. Furthermore, it was shown that treatment of BEAS-2B cells with exogenous type III IFN induced the expression of ISGs, e.g., CCL5 and CCL10, in resting cells, as well as after RV16 infection, in the latter case the induction of those genes was many times higher than in uninfected cells (65). As shown in the study by Wark et al., also here viral replication was increased in HBECs isolated from asthmatic subjects as compared to healthy controls, and here, it was observed that Type III-IFN expression was decreased in asthmatics. These results were then confirmed in primary bronchoalveolar lavage cells, where cells isolated from asthmatic patients and *ex vivo* treated with RV16 also secreted lower amounts of type III-IFN. To substantiate these findings, the same subjects from each cohort were experimentally infected with RV and clinical parameters were evaluated. It was shown that asthmatic patients experience a higher cold score, higher airway inflammation, and higher virus load, which inversely correlated with the decreased type III IFN secretion observed earlier in the *ex vivo* experiments, indicating a defect of patients suffering from asthma to mount effective antiviral responses against RV (65).

Another study using HBEC from healthy and asthmatic volunteers, which were then *in vitro* infected with either minor group RV1b or major group RV16, found that there was no difference in IFN-β or type III IFN secretion from both study groups after 48 h of culture after RV1b infection. However, *IFNL1* mRNA expression was even increased in HBECs from asthmatic patients after infection with RV16 and subsequent 48 h of cell culture. The release of viral particles from RV1b or RV16 infected cells from both study groups did not differ significantly at any time-point analyzed. An explanation for the contradictory results could be the use of HBEC from patients with different asthmatic phenotypes or might be due to technical differences (66).

However, a protective role of type III IFN in asthma has also been shown by a group in a study using a murine model of allergic asthma (19). In the study, they found that in mice lacking the IL-28 receptor α chain (IL–28Rα^–/–^) the asthmatic phenotype was worsened, accompanied by increased airway inflammation and mucus production, as well as higher levels of Th2- and Th17-associtated cytokine secretion. In contrast, wild type mice treated with either recombinant or adenoviral expressed IFN-λ2 (IL–28A) showed an improvement of asthmatic symptoms, concomitant with diminished Th2 and Th17 responses and an increase in IFN-γ expressing Th1 cells. This latter effect was attributed to lung CD11c^+^ DCs, which, in response to stimulation with IL-28A, downregulate the co-stimulatory molecule OX40 ligand (OX40L) and upregulate IL-12 production, a cytokine promoting Th1 cell development (Figure 3) (63).

In a recent study, our group has shown that in a cohort of pre-school children with and without asthma at baseline, *IFNA* mRNA levels were markedly decreased in PBMC isolated from the asthmatic group, both at mRNA level in PBMCs and at protein level in serum, when the children were sub-divided in accordance to RV detection in their upper airways. The analysis of type III IFN in serum instead revealed an increase of this cytokine in children with positive RV detection in their airways in both the control (tendency) and the asthmatic (significant) group. During the course of the study, asthmatic children were asked to come to their study center within 2 days, when they experience a respiratory infection/cold or an exacerbation of their disease (=symptomatic visit). When the baseline data of serum IFN-α were compared to those at symptomatic visits, a significant increase in IFN-α was observed in children with RV detected in their upper airways. However, at symptomatic visits, virus detection was positive in all children, so that no statement could be made about the RV negative group. In contrast, serum type III IFN levels did not significantly differ between the recruitment visit and symptomatic visit. This indicates that these responses are transient and dependent on certain stimuli (67).

Taken together, so far different results on the ability of asthmatic patients to respond to RV with IFNs have been reported. Probably, the time of analysis and the material analyzed is of importance to get significant results. Furthermore, as asthma is very heterogeneous, also the stage of disease or the underlying immunological processes might influence the detection of IFNs and thereby influence the conclusions drawn.

## Clinical Implications and Future Directions

Currently, the therapy of asthma is still mainly symptomatic with a combination of treatment with corticosteroids to inhibit the inflammatory processes in the lung and/or β2-agonists in order to relieve the bronchospasm (68). Recent therapeutic strategies focused on the neutralization of single effector molecules, such as IgE (omalizumab) and IL-9 (MEDI-528) or on receptor blockade, e.g., by a mutated form of IL-4 (pitrakinra), which binds to the IL-4Rα chain and blocks the binding of IL-4 and IL-13 [reviewed in Ref. (68)]. So far, these approaches did not bring a breakthrough in asthma therapy.

The use of IFN-α as a therapeutic agent for persistent RV infections has shown promising results, e.g., as RV RNA was cleared after subcutaneous administration to patients with hypogammaglobulinemia (69). However, in other trials treating either healthy volunteers intra nasally with IFN-α2 after experimental RV infection, or asthmatic patients with steroid-resistant disease, the symptoms were alleviated dose-dependently but side-effects such as headaches and nausea were also observed. Similar results were obtained after IFN-β administration, limiting the use of type I IFNs for asthma therapy [reviewed in Ref. (19, 70)]. As the action of type III IFNs is not as broad as that of type I IFNs, due to limited receptor distribution, their use might be advantageous in the treatment of different diseases. So far, few trials with type III IFN have been conducted in humans and many more details about the connection of different symptoms and IFN expression need to be clarified before these can be established. Yet, current data hint the potential of type III IFN administration as a therapeutic option for example for asthma, as it is able to modulate immune responses, e.g., by inhibiting Th2 and inducing Th1 responses (63, 71).

## Author Contributions

NS wrote the introduction and the IFN signal transduction part and generated Figure 2. AP and JK did the part of the virus infection and IFN-induction and generated Figure 1. SF supervised the all process enclosing the figures and manuscript.

## Conflict of Interest Statement

The authors declare that the research was conducted in the absence of any commercial or financial relationships that could be construed as a potential conflict of interest.
